# Supplementary material for: The effect of mothers and caregivers’ fasting status on the dietary diversity of children 6-23 months: A longitudinal study in Debrebirhan, Ethiopia
Source: PLoS One. 2022 Feb 24;17(2):e0264164. doi: 10.1371/journal.pone.0264164 (PMC8870544; doi:10.1371/journal.pone.0264164)
Supplement: Attachment — Submitted filename: PONE_Review_210916.pdf [file pone.0264164.s002.pdf]

Thank you for kindly providing the opportunity to review the review.

The authors present an original community-based research study in Ethiopia that quantitatively assesses the effect of religious-based fasting on the dietary patterns of children. The authors conducted longitudinal data collection from urban and rural households in two rounds to capture pre fasting and post fasting periods from mothers of approx. 218 children **aged 6-23 months** in 2019. The majority (71%) of infants and young children were under 17 months(one and half years of age), with 89 % of mothers reporting that they were breastfeeding at the time of data collection. While the study describes fasting within one community in Ethiopia, Orthodox Christians, who routinely fast during the year in terms of levels /types of food abstinence, the younger age of the study cohort and socio-economic characteristics can be expected to strongly influence the study findings. The paper uses McNemars paired test to present pre and post fasting changes on daily dietary intake for children with results showing a reduction of daily recommended intake in children during fasting. The study classifies fasting status in the mothers according to the specific daily time till when the mother had fasted on the previous day , eg fasted till 9 am, 3 pm 6pm etc. While the study presents an interesting perspective, and the method adequately provides insights, the relatively small sample size and specific study characteristics impact the publication potential of the paper. The paper could have produced stronger results instead if they used regression analysis to account for the change in dietary intake in young children while accounting for common confounders income, education and parity. The presentation of findings in table 3(line 215) does not explain the claims made in the discussion/conclusion(lines 309). The expression used is sufficiently clear, but the references have multiple formatting issues

The study still highlights a novel research topic that has broader social implications, especially for young infants whose health is sensitive to nutritional changes surrounding cultural practices. I would therefore consider the paper for publication after the authors address the

comments below and other reviewers. There are various issues that I have raised below for the authors to first address, which are multiple but clustered around the interpretation of the findings.

**Methodology:**

**Question:** While study setting details are provided in line 115, it was not clear whether the same households were visited in both rounds. The study findings seem to suggest that, however, a clearer description is required.

**Comment** Additionally, the study setting section also requires details to explain the rationale for selecting 'Debrebirhan woreda' as well as the importance of the north Shewa zone. The authors may like to provide some lines providing a description of the geographical administrative hierarchy of the study setting.

**Results**

**Question**: Please consider descriptive formatting table(1) lines 175-176 to one page for ease in reading.

**Question**: The fasting characteristics as provided in table 2 are informative but limit interpretation of fasting status to readers not familiar to the unique social-cultural aspects of the Ethiopian study population. For example, the line describing- *'Not consuming animal source food including fish and fast up to 9 am'/ Not consuming animal source food including fish and fast up to 12 pm'* do not communicate information commonly understood and applied.

**Comment** : The authors may have liked to develop an index of fasting such as done for religiosity that is commonly done in the broader literature assessing impact of religion on nutrition instead of using time frames to separate fasting behaviours. A fasting index also allows for additional methods such as regression to undertake the study.

**Comment:**I am concerned with the results explaining changes in dietary diversity among the samples households (page 16,line 197-199) without adequately accounting for levels of household income, education and parity. These are established social determinants that have a strong influence on a household's purchasing capacity and decision making. I would request the authors to share why regression analysis was not considered as an appropriate method for the paper.

**Question**: As the sampled children comprise of young infants and children aged 6 months-23 months with almost 71 % of total sample (table 1/child age in months) under the age of 17 months, the effect of fasting on dietary diversity as produced in lines 200-205 needs to consider the fact that most young children may still be breastfed which may not be affected much as a practice during fasting

**Grammatical error**-The following  lines 240-242 are missing information- '*We a significant difference in dietary diversity due to change  in the consumption of meat, egg, milk and Vitamin A rich fruits and vegetables'*

**Discussion:**

**Question** While the study found a reduction in animal sources of food (line 240-242) during fasting; families are also potentially constrained in buying animal sources of protein during periods where food pricing becomes a barrier. In the study context, can the authors assess if previous literature provides information on changes to food costs during fasting. Such findings would be useful for the authors to consider and report on as a potential factor impacting some of their study findings.

**Question**

In the absence of controlling for income, or parity, or number of siblings, the author may like to reconsider the current interpretation presented in lines 262-265

**Conclusion**

The claims made in lines 309-311 regarding reasons(contamination of utensils) why mothers may be limiting consumption of animal source foods is probable but have not been investigated in the study. There are no results to support the claim; thus the authors are advised to correct the current interpretation/reasoning.

Lines 315. I would have also liked to see the authors providing some examples of potential intervention strategies that could be applied in the study population. The potential of informing mothers of dietary changes to the children and potential health impacts is important from a public health perspective.

**Reference**

Multiple References have formatting errors such as ref 8( incorrect format about editor) 9( abbreviation should be expanded). 13 organisation is incorrectly formatted(*13. Thrive. A. IYCF Practices,)  ref 16(incorrect format)*
