# Supplementary material for: The effect of mothers and caregivers’ fasting status on the dietary diversity of children 6-23 months: A longitudinal study in Debrebirhan, Ethiopia
Source: PLoS One. 2022 Feb 24;17(2):e0264164. doi: 10.1371/journal.pone.0264164 (PMC8870544; doi:10.1371/journal.pone.0264164)
Supplement: Attachment — Submitted filename: Response to reviewers.docx [file pone.0264164.s003.docx]

***Response to Reviewer 1***

**Thank you for your review of our paper. We have answered each of your points below**

**Formatting Comments:** From line 38 to 311

**Response**: Thank you so much for the comments and edits! We found it very helpful.

**General comments**
Generally, the authors tried to describe the effect of mothers/caregivers’ fasting status on the dietary diversity of children 6-23months in the study area.
However, in few places within the manuscript, authors started sentences with figures, this should be corrected. To improve clarity, the manuscript will benefit from language editing.

**Response**: Thank you for your comment. We have actually used Mrs. Heather Moorman, a language editor, and we would like to highly acknowledge her for her assistance. heather.c.moorman@gmail.com

***Response to Reviewer 2***

**Thank you for your review of our paper. We have answered each of your points below**

**Comment**: All the justifications are in place. However, I think the author has to add some points to strong support the rationale for conducting this study which will add value to the research. This will help further research on this area.

**Response**: Thank you very much for your comment, we have found it very helpful

The best rationale for this study is that various literatures have focused on other factors related to the child, mothers or household characteristics; we only got limited number of papers that took into account the impact of religious beliefs or fasting on child dietary diversity by which their impact could be greater in Ethiopia, where people are very attached to their religion. This is especially works for Orthodox Christian followers, who make up nearly half of the country's population.

Few studies have been conducted that considers religious fasting periods as one factor to influence mothers' feeding practices, particularly when preparing animal source foods. (1, 2) But those studies have limitations: they both did not measure child dietary diversity during the fasting and non-fasting periods, didn’t quantify the difference in child dietary diversity in these two periods, or didn’t control the mother’s fasting status in their analysis, so it is difficult to draw conclusions from those studies. As a result, we conducted a before-and-after study on the same mothers with children aged 6 to 23 months, measuring MDD during the non-fasting period first and then during the fasting period later on; we compared and magnified the difference in the child's dietary diversity between these two periods. Individual, child, and HH factors were not confounding for this study because we compared intraindividual differences

***Response to Reviewer 3***

**Thank you for your review of our paper. We have answered each of your points below**

**Methodology:**

**Question:** While study setting details are provided in line 115, it was not clear whether the same households were visited in both rounds. The study findings seem to suggest that, however, a clearer description is required.

**Response**: Thank you for your comment. Yes, the same households/participants were interviewed both before and after the fasting season. This is mentioned in line 148 of the study procedure, but as per your suggestion we have also now included it on Line 124 of the study setting section

**Comment** Additionally, the study setting section also requires details to explain the rationale for selecting ‘Debrebirhan woreda’ as well as the importance of the north Shewa zone. The authors may like to provide some lines providing a description of the geographical administrative hierarchy of the study setting.

**Response**: We acknowledge your comment. According to the Ethiopian Demographic and Health Survey (EDHS) of 2016, the Amhara region has the lowest Children MDD, next to Afar region, and we randomly selected North Shewa Zone Debrebirihan Wereda from the Amhara region

**Results**

**Question**: Please consider descriptive formatting table(1) lines 175-176 to one page for ease in reading.

**Response**: Thank you for your comment. We have incorporated your comment on the revised manuscript

**Question**: The fasting characteristics as provided in table 2 are informative but limit interpretation of fasting status to readers not familiar to the unique social-cultural aspects of the Ethiopian study population. For example, the line describing- *‘Not consuming animal source food including fish and fast up to 9 am’/ Not consuming animal source food including fish and fast up to 12 pm’* do not communicate information commonly understood and applied.

**Response**: Thank you for your feedback. According to Ethiopian Orthodox dogma, people can fast from eating animal source foods, abstain from eating animal foods, and even go for several hours without eating anything. As a result, the fasting status of one person may differ from that of another depending on the length of fasting. Fasting generally entails eating only one meal per day, either in the evening or after 2.45 p.m., and abstaining from meat, fats, eggs, and dairy products. However, some adhere strictly to this fasting rule, while others can fast (abstain from eating/drinking anything) for a few hours (9am or 12pm, for example), or some only fast from eating animal foods but may not stay up for several hours without taking any foods/drinks. That is why we categorized the fasting status and inquired about the time period. But we appreciate the comment and we have put this as a description or as an operational definition in the study procedure/measurment

**Comment** : The authors may have liked to develop an index of fasting such as done for religiosity that is commonly done in the broader literature assessing impact of religion on nutrition instead of using time frames to separate fasting behaviors. A fasting index also allows for additional methods such as regression to undertake the study.

**Response**: Thank you for your feedback: Yes, we used a repeated measurement on the same individuals, which we assumed would have a stronger interpretation from the study design standpoint. But, as you suggest, it is also possible to measure the effect of fasting on child MDD using only one time period a (during fasting time) and using regression analysis to control for potential confounders. As a result, we have included this in the recommendation section for future studies to consider.

**Comment:** I am concerned with the results explaining changes in dietary diversity among the samples households (page 16,line 197-199) without adequately accounting for levels of household income, education and parity. These are established social determinants that have a strong influence on a household’s purchasing capacity and decision making. I would request the authors to share why regression analysis was not considered as an appropriate method for the paper.

**Response**: Thank you! We found your comment helpful.

As you can see in the table below, the goal of this study is to see how fasting affects children's dietary diversity by measuring dietary diversity of children aged 6 to 23 months in the same household; children’s dietary diversity before and during the fasting season (kindly see the below table). So, because we measured the outcome variable twice on the same participants, the sociodemographic variables are usually static and will not affect intraindividual comparison. We compared the dietary diversity of a single child before and during the fasting period, not the dietary diversity of one child with other children. As a result, the study is a repeated measurement on the same households, and other variables may not need to be controlled when comparing the same individuals. This is the study's initial assumption

| MDD Before fasting | MDD During fasting |
| --- | --- |
| Child A | Child A |
| Child B | Child B |
| Child C | Child C |

**Question**: As the sampled children comprise of young infants and children aged 6 months-23 months with almost 71 % of total sample (table 1/child age in months) under the age of 17 months, the effect of fasting on dietary diversity as produced in lines 200-205 needs to consider the fact that most young children may still be breastfed which may not be affected much as a practice during fasting

**Response**: Thank you for your comment***.*** We definitely agree that age is an identified factor for dietary diversity and can also influence the effect of fasting on it, but as we tried to emphasize above, the focus of this study was to compare dietary diversity on the same participants (Child A with Child A, etc.) but on different time period (Before the lent fast season/Non fasting period AND during lent fast season/Fasting period). However, we can include this as a limitation so that other studies can address it.

**Grammatical error**-The following lines 240-242 are missing information- ‘*We a*

*significant difference in dietary diversity due to change in the consumption of meat, egg, milk and Vitamin A rich fruits and vegetables’*

**Response**: Thank you! We found your comment helpful and have revised accordingly on line 247-248

# Discussion:

**Question** While the study found a reduction in animal sources of food (line 240-242) during fasting; families are also potentially constrained in buying animal sources of protein during periods where food pricing becomes a barrier. In the study context, can the authors assess if previous literature provides information on changes to food costs during fasting. Such findings would be useful for the authors to consider and report on as a potential factor impacting some of their study findings.

**Response**: Thank you for your comment***.*** Yes, food price is another seasonal variable influenced by religious fasting in Ethiopia. However, according to the literature, animal-source foods will be cheaper during fasting periods because most orthodox Christians will not consume these foods. So, during fasting periods, meat, milk, eggs, and so on will be more readily available and cheaper. So, consumption of animal source foods were supposed to be higher and more readily available during fasting periods, but what really mattered was the mothers' fasting situations and the finding was the opposite. Animal source food consumption decreased during fasting period (ref)

# Question

In the absence of controlling for income, or parity, or number of siblings, the author may like to reconsider the current interpretation presented in lines 262-265

**Response**: Thank you for your comment. We will recommend this for other studies to control in a regression analysis while conducting additional research.

# Conclusion

The claims made in lines 309-311 regarding reasons (contamination of utensils) why mothers may be limiting consumption of animal source foods is probable but have not been investigated in the study. There are no results to support the claim; thus the authors are advised to correct the current interpretation/reasoning.

**Response**: Thank you! We found your comment helpful and have revised the language accordingly

Lines 315. I would have also liked to see the authors providing some examples of potential intervention strategies that could be applied in the study population. The potential of informing mothers of dietary changes to the children and potential health impacts is important from a public health perspective.

**Response**: Thank you for your comment. Based on our findings, potential intervention strategies include nutrition education at a church using religious people and nutrition education at a health facility using health care providers for mothers with children aged 6 to 23 months of age. Mothers should be informed that fasting seasons or their own fasting status should not have an impact on their children's feeding practices.

# Reference

Multiple References have formatting errors such as ref 8(incorrect format about editor) 9(abbreviation should be expanded). 13 organization is incorrectly formatted (*13. Thrive. A. IYCF Practices,) ref 16(incorrect format)*

**Response**: Thank you for your comment. We have corrected this accordingly

**Additional Comments**

We suggest you thoroughly copyedit your manuscript for language usage, spelling, and grammar. If you do not know anyone who can help you do this, you may wish to consider employing a professional scientific editing service.

**Response**: We used Mrs. Heather Moorman, a language editor, and we would like to highly acknowledge her for her assistance. heather.c.moorman@gmail.com

**Comment**

a. Please clarify the sources of funding (financial or material support) for your study. List the grants or organizations that supported your study, including funding received from your institution.

**Response**: Thank you for your comment. We received funding for data collection from Addis Ababa University's School of Public Health, but as stated, the funders had no role in study design, data collection and analysis, publication decision, or manuscript preparation.

b. State what role the funders took in the study. If the funders had no role in your study, please state: “The funders had no role in study design, data collection and analysis, decision to publish, or preparation of the manuscript.”

**Response**: Thank you for your comment. We will include this statement in the manuscript, “The funders had no role in study design, data collection and analysis, publication decision, or manuscript preparation”

c. If any authors received a salary from any of your funders, please state which authors and which funders.

**Response**: Thank you for your comment

d. If you did not receive any funding please state: “The authors received no specific funding for this work.

**Response**: Non of the authors received a salary from the funder for this study,

*Reference*
