# Supplementary material for: The effect of mothers and caregivers’ fasting status on the dietary diversity of children 6-23 months: A longitudinal study in Debrebirhan, Ethiopia
Source: PLoS One. 2022 Feb 24;17(2):e0264164. doi: 10.1371/journal.pone.0264164 (PMC8870544; doi:10.1371/journal.pone.0264164)
Supplement: Attachment — Submitted filename: Response to Reviewers.docx [file pone.0264164.s004.docx]

***Response to Reviewers***

**Thank you very much for your review of our paper.**

**Comment:** Upon reviewing this manuscript myself, there remain some minor language/editing issues throughout

**Response:** Thank you so much for the comments! We found it very helpful and have revised the language for some of manuscript section accordingly

**Comment**: Please also ensure that all terms which may be unfamiliar to readers are defined, such as 'woreda' and 'kebele

**Response**: Thank you for your comment, we have put the definitions for both in the sentence (Line 120 to 122)

**Comment**: Please also take care to improve statistical reporting and report exact p-values for all values greater than or equal to 0.001, and refer to p-values as "p<.001" instead of "p=.000".

**Response**. We appreciate your feedback and thank you so much or sharing the link for the PLOS statistical report guideline, we found it helpful. All the p values related to the main outcome were found to be less than 0.001, so that is why we put as P<0.001
